# Erythropoiesis-stimulating agent hyporesponsiveness and malignancy development in patients with non-dialysis chronic kidney disease: a prospective cohort study

**DOI:** 10.1007/s10157-025-02769-7

**Published:** 2025-10-27

**Authors:** Nobuhiro Hashimoto, Terumasa Hayashi, Tatsuo Kagimura, Ichiei Narita

**Affiliations:** 1 Department of Kidney Disease and Hypertension, Osaka General Medical Center, Osaka, 558-8558 Japan; 2 The Translational Research Center for Medical Innovation, Hyogo, Japan; 3 Graduate School of Medical and Dental Sciences, Niigata University, Niigata, Japan

**Keywords:** Erythropoiesis-stimulating agents, Erythropoiesis-stimulating agents hyporesponsiveness, Chronic kidney disease, Malignancy

## Abstract

**Background:**

Erythropoiesis-stimulating agents (ESA) hyporesponsiveness may be linked to malignancy, but studies examining this association are limited. We investigated whether initial ESA hyporesponsiveness and changes in responsiveness may serve as clinical markers reflecting undiagnosed malignancy in patients with non-dialysis-dependent chronic kidney disease (NDD-CKD).

**Methods:**

We used data from the BRIGHTEN, a prospective study of NDD-CKD patients with anemia. Initial ESA responsiveness was assessed using the erythropoietin resistance index (ERI-1B), calculated as the ratio of darbepoetin-alfa dose (μg) to hemoglobin concentration (g/dL) at 12 weeks after darbepoetin-alfa initiation. ESA responsiveness trends after 12 weeks were analyzed using a joint latent class model (JLCM). The associations of both initial ESA responsiveness and ESA responsiveness trends after 12 weeks with malignancy development were analyzed using a Cox proportional hazards model.

**Results:**

Of the 1641 patients analyzed, 44 developed new malignancies. Patients with poor ESA response at 12 weeks (ERI-1B > 3.8 μg/g/dL) had a higher incidence of malignancy compared to those with better ESA response (adjusted hazard ratio [HR]: 2.07; 95% confidence interval [CI]: 1.07–4.00). Furthermore, based on the JLCM, patients in the poor response group, characterized by a faster decline in ESA responsiveness after 12 weeks, had a higher risk of malignancy than the good response group (adjusted HR: 2.01; 95% CI: 1.08–3.72).

**Conclusion:**

Both initial ESA hyporesponsiveness and subsequent declines in responsiveness were significantly associated with the development of malignancy in patients with NDD-CKD. ESA hyporesponsiveness may serve as a clinical marker that reflects an increased risk of undiagnosed malignancy.

**Supplementary Information:**

The online version contains supplementary material available at 10.1007/s10157-025-02769-7.

## Introduction

The introduction of erythropoiesis-stimulating agents (ESAs) has significantly improved the management of anemia in patients with chronic kidney disease (CKD). However, using ESAs to raise hemoglobin (Hb) levels to the normal range has been found to worsen prognosis [1–5]. Moreover, conditions leading to ESA hyporesponsiveness may adversely affect outcomes [6–10]. Therefore, ESA hyporesponsiveness has been recognized as a significant predictor of adverse prognosis, highlighting the importance of understanding and addressing this condition in clinical practice [8, 11–13].

The association between ESA hyporesponsiveness and malignancy might be commonly perceived as well-established, given that malignancy is listed in various guidelines as a potential cause of ESA hyporesponsiveness [11, 13]. However, few reports have examined the relationship between ESA hyporesponsiveness and malignancy. The Trial to Reduce Cardiovascular Events with Aranesp Therapy (TREAT) study found higher cancer-related deaths in patients with a history of malignancy in the ESA-treated group than in the placebo group; however, it did not directly assess the link between ESA hyporesponsiveness and malignancy [4]. The rationale behind the inclusion of malignancies as a cause of ESA hyporesponsiveness in various guidelines is not entirely clear. This might be based on general population studies showing that 30% of patients were anemic at the time of malignancy diagnosis [14] and on clinical observations in which some patients with ESA resistance were later diagnosed with malignancy.

Given the limited evidence on this topic, we aimed to evaluate whether ESA hyporesponsiveness may serve as a clinical marker of subsequently diagnosed malignancies in patients with non-dialysis-dependent CKD (NDD-CKD) using data from the oBservational clinical Research In chronic kidney disease patients with renal anemia: renal prognosis in patients with Hyporesponsive anemia To Erythropoiesis-stimulating agents, darbepoetin alfa (BRIGHTEN).

## Materials and methods

This study used data from the BRIGHTEN study, whose protocol has been described in detail elsewhere [15]. Briefly, the BRIGHTEN study was a prospective, observational study of patients with NDD-CKD who presented with anemia (Hb < 11 g/dL). Patients with a history of ESA treatment, hematological disease, bleeding lesions, or active malignancies were excluded. Patients were enrolled between May 2014 and March 2016 and followed up for 144 weeks: 96 weeks of darbepoetin-alfa (DA) administration, after which patients were free to receive additional treatments as needed. The study was conducted in accordance with the principles of the Declaration of Helsinki and the Ethical Guidelines for Medical and Health Research Involving Human Subjects issued by the Ministry of Health, Labor and Welfare of Japan. Written informed consent was obtained from all participants before enrollment. The protocol was approved by the central institutional review board (Nagoya University, No. 2014–0027) and by the ethics committees of all participating institutions.

### Definition of the occurrence of new malignancies and follow-up

Outcome was defined as the occurrence of new malignancies within 48 weeks from the end of the 96-week DA administration period. The presence and type of malignancy were evaluated every 12 weeks by site investigators at each participating institution. Investigators reviewed comprehensive medical records, including relevant laboratory data, imaging studies, and pathology reports when available, to document any newly diagnosed malignancies. The diagnostic approach was determined by the judgment of site investigators and reflected routine clinical practice in Japan, rather than being based on a centralized adjudication process. Patients were followed from study baseline until death, loss to follow-up, dialysis initiation, or the end of the observation period, whichever came first. If a malignancy occurred, the patient was excluded from further analysis.

### Indicators of ESA responsiveness

We used the erythropoietin resistance index (ERI-1B), calculated as the DA dose (μg) divided by the Hb concentration (g/dL), as an indicator of ESA responsiveness, given that this index has been most associated with prognosis in previous reports [16].

### Statistical analysis

Baseline characteristics are reported as means ± standard deviation (SD), median (interquartile range [IQR]), or number (percentage).

### Relationship between initial ESA response and the occurrence of new malignancies

The 12-week ERI-1B after DA initiation was used to draw a time-dependent receiver operating characteristic (ROC) curve for the development of malignancies within 2 years, and the optimal cutoff value for predicting subsequent malignancies was determined using the closest-to-(0,1) method. The patients were divided into two groups according to the cutoff values obtained. The predictive relationship between ESA responsiveness and the subsequent occurrence of new malignancies was evaluated using a Cox proportional hazards model, adjusted for age, history of malignancy, smoking habits, diabetes status, baseline ferritin levels, high-sensitive C-reactive protein, serum albumin, and estimated glomerular filtration rate. Cumulative survival was estimated using the Kaplan–Meier method and compared using the log-rank test, and the yearly incidence rate was estimated.

### Relationship between ESA responsiveness trends and the occurrence of new malignancies

A joint latent class model (JLCM) implemented in the “lcmm” R package was used to estimate the relationship between trajectories of ERI-1B measured from 12 to 96 weeks after the start of DA administration and the development of malignancies. The JLCM assumes that the population of participants is heterogeneous and comprises homogeneous latent subgroups that share the same trajectory of a repeated measure and the same risk of event occurrence. Using a linear mixed model, we modeled the trajectory of a repeated measure, ERI-1B. The mean trajectory of the repeatedly observed ERI-1B was estimated using restricted cubic spline curves, which allowed for the consideration of nonlinear changes in ERI-1B. The linear mixed model was then combined with the proportional hazard model via the multinomial logistic regression model, known as the JLCM. In the linear mixed model, we assumed that the parameters were independent of the trajectory classes and did not include covariates other than the measurement points. For descriptive purposes, the longitudinal change in ERI-1B within each latent class was also summarized using a slope, defined as the weekly change in ERI-1B from 12 to 96 weeks. In the proportional hazard model, we specified the event as the development of the malignancies defined above and added age, history of malignancy, smoking habits, diabetes status, baseline ferritin levels, high-sensitive C-reactive protein, serum albumin, and estimated glomerular filtration rate as covariates. The models with one to six classes were fitted using a grid search, and the model with two classes was selected based on the Bayesian Information Criterion, the posterior probability of membership. After fitting the model, the baseline characteristics of the patients in each class were compared. Moreover, the mean trajectory of the observed ERI-1B was plotted to determine whether there was any divergence between the estimated mean trajectory and the observed mean. Moreover, the cumulative incidence of the development of malignancies was estimated. The ERI-1B adjusted mean over time was estimated using the mixed-effects model with repeated measurements (MMRM) with a marginal model that had a common variance–covariance structure between time points.

As a sensitivity analysis, a similar JLCM analysis was performed on the trajectory of ERI-1B up to 30 and 60 weeks after administration of the DA preparation and compared the results.

All analyses were performed using R 4.1.3. A P-value of < 0.05 with two sides was considered statistically significant.

## Results

### Patients

**Fig. 1:**
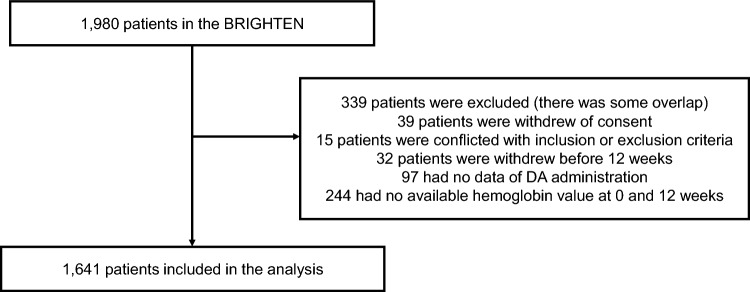
Flow diagram of the study participants, DA, darbepoetin alfa

Figure 1 presents a flowchart of the study. Of the 1,980 patients enrolled in the BRIGHTEN study, 1,641 were analyzed. Baseline characteristics have been described in detail elsewhere [17]. The mean age was 70 ± 12 years, and 59% of the patients were male. There were 179 current smokers (11%), 590 ever-smokers (36%), and 200 individuals with a history of malignancy (12%). Forty-four patients developed new malignancies. Table 1 presents a list of the new malignancies. Colorectal cancer was the most common (9 patients), followed by gastric cancer (6 patients) and prostate cancer (4 patients).

**Table 1:** List of new malignancies

Types of malignancy	*N* = 44
Colorectal cancer	9
Stomach cancer	6
Prostate cancer	4
Lung cancer	3
Bladder cancer	3
Liver cancer	2
Laryngeal cancer	2
Malignant lymphoma	3
Skin cancer	3
Esophageal cancer	1
Renal and urinary tract cancer	1
Gallbladder cancer	1
Thyroid cancer	1
Parathyroid cancer	1
Myelodysplastic syndrome	1
Tongue cancer	1
Submucosal tumors	1
Adenocarcinoma	1

### Relationship between initial ESA response and the occurrence of new malignancies

**Fig. 2:**
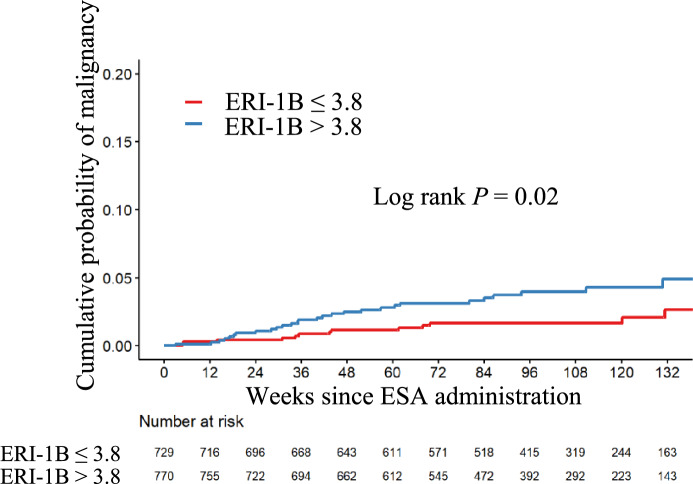
ESA responsiveness stratified by the 12-week ERI-1B after DA initiation and incident malignancy, Patients with ERI-1B > 3.8 had a significantly higher incidence of malignancy compared to those with ERI-1B ≤ 3.8 (log-rank *P* = 0.02). ESA, Erythropoiesis-stimulating agents; ERI, erythropoietin resistance index

The optimal ERI-1B cut-off value at 12 weeks after DA initiation for predicting subsequent malignancy development was identified as 3.8 μg/g/dL based on a time-dependent ROC curve analysis. The group with an initial low ESA response (ERI-1B > 3.8 μg/g/dL) had a higher incidence of malignancy compared to the good response group (ERI-1B ≤ 3.8 μg/g/dL), with an adjusted hazard ratio (HR) of 2.07 (95% confidence interval [CI], 1.07–4.00) (Table 2). The incidence of malignancy was 2.07 per 100 person-years (95% CI, 1.38–2.99) in the low response group, compared to 0.96 (95% CI, 0.51–1.64) in the good response group (Table 2). The cumulative incidence curve is shown in Fig. 2.

**Table 2:** Initial ESA response and the occurrence of new malignancies

	Number of patients*	Number of new malignancies	Incident rate (/100 Pearson years) (95% CI)	Hazard ratio (95% CI)	Adjusted Hazard ratio (95% CI) †
ERI-1B ≤ 3.8	729	13	0.96 (0.51–1.64)	Ref	Ref
ERI-1B > 3.8	770	28	2.07 (1.38–2.99)	2.14 (1.11–4.13)	2.07 (1.07–4.00)

ERI-1B was calculated as [DA dose (μg)/Hemoglobin concentration (g/dL)] at 12 weeks after darbepoetin alfa initiation

^*^ Those who could not obtain ERI-1B at 12 weeks after darbepoetin-alfa initiation were excluded

^†^ Analyses were adjusted for age, history of malignancy, smoking habits, diabetes status, baseline ferritin levels, high-sensitive C-reactive protein, serum albumin, and estimated glomerular filtration rate

*ESA* Erythropoiesis-stimulating agent, *ERI* erythropoietin resistance index, *CI* confidence interval

### Relationship between ESA responsiveness trends and the occurrence of new malignancies

**Fig. 3:**
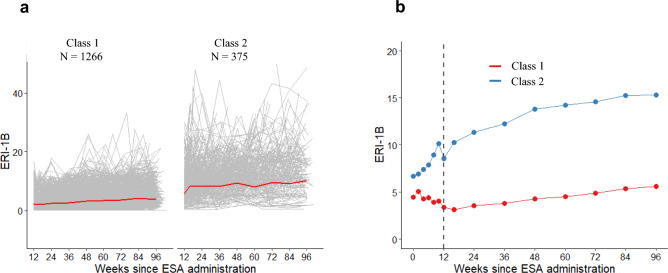
Trajectories of ERI-1B in the two latent classes, **a** Trajectories of ERI-1B from 12 weeks after the initiation of ESA administration to the end of ESA administration (96 weeks). The gray lines indicate ERI-1B trends for individual cases. The red line indicates the average ERI-1B value. **b** Changes in the adjusted mean ERI-1B values using the MMRM method in the two latent classes from the initiation of ESA administration to the end (96 weeks). The changes from the initiation of ESA administration to 12 weeks are not reflected in the class classification. ERI, erythropoietin resistance index; ESA, erythropoiesis-stimulating agents

A model with two latent classes was selected for analysis. Class 1 (good response group) comprised 1266 patients (77%) and was characterized by better ESA responsiveness at 12 weeks, with a mean ERI-1B at 12 weeks of 3.3 ± 2.3 μg/g/dL, followed by a very slow decline in responsiveness thereafter (slope: 0.030 μg/g/dL/week, 95% CI, 0.025–0.036) (Fig. 3). Class 2 (poor response group) included 375 patients (23%) and was characterized by poor ESA responsiveness at 12 weeks, with a mean ERI-1B at 12 weeks of 8.6 ± 5.8 μg/g/dL, followed by a faster decline in responsiveness than in Class 1 (slope: 0.106 μg/g/dL/week, 95% CI: 0.095–0.117) (Fig. 3).

**Fig. 4:**
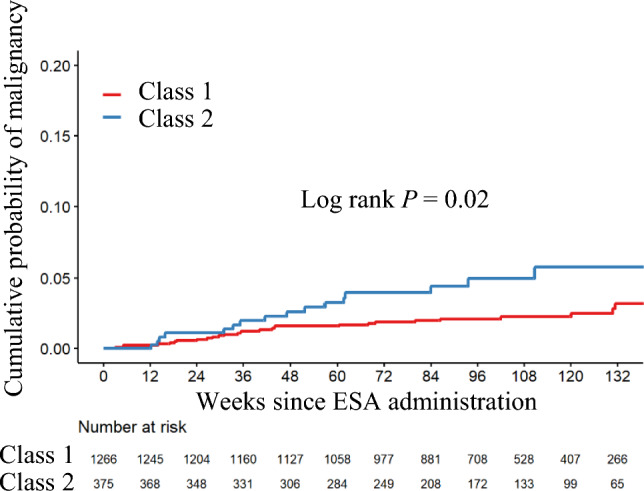
ESA responsiveness stratified by class of ERI-1B trajectory and incident malignancy, Class 2 patients (poor response group) had a significantly higher incidence of malignancy than Class 1 patients (good response group) (log-rank, *P* = 0.02). *ESA* Erythropoiesis-stimulating agents, *ERI* erythropoietin resistance index

Table 3 shows the baseline characteristics stratified by ERI-1B trajectory patterns. Class 1 had a lower prevalence of smoking habits and fewer individuals with a history of malignancy compared to Class 2. Class 2 had a higher incidence of malignancy than Class 1, with an adjusted HR of 2.01 (95% CI, 1.08–3.72) (Table 4). The incidence of malignancy was 2.54 per 100 person-years (95% CI, 1.45–4.12) in Class 2, compared to 1.20 (95% CI, 0.80–1.73) in Class 1 (Table 4). The cumulative incidence curve is shown in Fig. 4. In Class 2, there was a higher incidence of colorectal cancer (Supplemental Table S1).

**Table 3:** Demographic and clinical characteristics by class

Characteristics	Total (*N* = 1641)	Class 1 (good response group) (*N* = 1266)	Class 2 (poor response group) (*N* = 375)	*p* value
*Demographics*				
Age years	69.9 ± 11.9	69.8 ± 11.8	70.2 ± 12.3	0.57
Male gender n (%)	962 (58.6)	733 (57.9)	229 (61.1)	0.30
Body mass index kg/m^2^	23.2 ± 4.1	23.0 ± 4.0	23.6 ± 4.4	0.03
Smoking status n (%)				0.04
Current	179 (10.9)	134 (10.6)	45 (12.0)	
Ever	590 (36.0)	438 (34.6)	152 (40.5)	
Never	806 (49.1)	637 (50.3)	169 (45.1)	
Unknown	66 (4.0)	57 (4.5)	9 (2.4)	
Malignancy (past history) n (%)	200 (12.2)	141 (11.1)	59 (15.7)	0.02
Comorbidities				
Hypertension n (%)	1,547 (94.3)	1,193 (94.2)	354 (94.4)	1.00
Diabetes n (%)	706 (43.0)	532 (42.0)	174 (46.4)	0.15
Dyslipidemia n (%)	902 (55.0)	698 (55.1)	204 (54.4)	0.85
Ischemic heart disease n (%)	263 (16.0)	202 (16.0)	61 (16.3)	0.95
Heart failure n (%)	116 (7.1)	85 (6.7)	31 (8.3)	0.36
Stroke n (%)	191 (11.6)	152 (12.0)	39 (10.4)	0.45
*Medications*				
RAS inhibitor use n (%)	1,069 (65.1)	827 (65.3)	242 (63.5)	0.83
Hypoglycemic agent use n (%)	522 (31.8)	395 (31.2)	127 (32.9)	0.36
Iron supplementation n (%)	233 (14.2)	171 (13.5)	62 (16.5)	0.19
*Laboratory values*				
Hemoglobin g/dL	9.8 ± 0.9	9.9 ± 0.8	9.3 ± 1.0	< 0.001
Mean corpuscular volume fL	93.1 ± 5.6	93.1 ± 5.4	93.1 ± 6.3	0.91
eGFR mL/min/1.73m^2^	20.1 ± 9.8	20.5 ± 9.6	18.7 ± 10.1	0.001
Albumin g/dL	3.7 ± 0.5	3.8 ± 0.5	3.6 ± 0.5	< 0.001
Ferritin ng/mL	134 ± 132	132 ± 128	143 ± 146	0.14
Transferrin saturation %	27 ± 10	27 ± 9	27 ± 11	0.69
High-sensitive C-reactive protein ng/ml	575 [218–1780]	557 [205–1650]	614 [280–2540]	0.003
Folic acid ng/mL	10.7 ± 34.9	10.0 ± 24.8	13.0 ± 57.0	0.15
Vitamin B12 pg/mL	414 ± 232	410 ± 227	429 ± 248	0.18
Urinary protein creatinine ratio g/gCr	2.3 ± 2.9	2.1 ± 2.8	3.0 ± 3.1	< 0.001

*RAS* renin-angiotensin system, *eGFR* estimated glomerular filtration rate

**Table 4:** ESA responsiveness trends and the occurrence of new malignancies

	Number of patients	Number of new malignancies	Incident rate (/100 Pearson years) (95% CI)	Hazard ratio (95% CI)	Adjusted Hazard ratio (95% CI) †
Class 1 (good response group)	1266	28	1.20 (0.80–1.73)	Ref	Ref
Class 2 (poor response group)	375	16	2.54 (1.45–4.12)	2.09 (1.13–3.86)	2.01 (1.08–3.72)

Classification was based on the joint latent class mixed model using the ERI-1B transition pattern 12 weeks after ESA administration

ERI-1B was calculated as [DA dose (μg)/Hemoglobin concentration (g/dL)]

^†^Analyses were adjusted for age, history of malignancy, smoking habits, diabetes status, baseline ferritin levels, high-sensitive C-reactive protein, serum albumin, and estimated glomerular filtration rate

*ESA* Erythropoiesis-stimulating agent, *ERI* erythropoietin resistance index, *CI* confidence interval

### Sensitivity analysis

The duration of administrative censoring for the JLCM was changed from 96 to 36 or 60 weeks. Two latent classes were selected for both analyses. As in the main analysis, Class 1 demonstrated better ESA responsiveness at 12 weeks, with a gradual decline in responsiveness thereafter. In contrast, Class 2 showed poorer ESA responsiveness at 12 weeks, with a faster decline in responsiveness thereafter (Supplemental Fig. S1 and S2). The cumulative incidence curve is shown in Supplemental Fig. S3 and S4. Class 2 was approximately twice as likely to develop malignancy compared to Class 1 (Supplemental Tables S2 and S3).

## Discussion

Our study demonstrated that initial ESA hyporesponsiveness 12 weeks after the start of ESA therapy was significantly associated with the subsequent development of malignancy. Moreover, a subsequent decline in responsiveness was also significantly associated with the development of malignancy. These findings suggest that ESA hyporesponsiveness may serve as a useful clinical marker that warrants attention, as it could reflect an underlying condition such as an undiagnosed malignancy. While these findings are in line with existing guidelines, this study is among the few to specifically examine the relationship between ESA responsiveness and the development of malignancy.

The KDIGO guidelines state that when an initial or subsequent ESA hyporesponsiveness occurs, a strong search for a cause, especially a correctable cause, should be undertaken [13]. Malignancy is listed as a cause, along with iron, vitamin B12, and folate deficiency, hypothyroidism, inflammation, hemolysis, bleeding, hyperparathyroidism, pure red cell aplasia, and malnutrition. In this study, the incidence of malignancy in the initial and subsequent hyporesponsive groups was approximately 2 to 2.5 per 100 person-years. While it is difficult to definitively assess whether this rate is high or low owing to the lack of comparable data, it is plausible that age could significantly influence this figure, as the mean age of our cohort is 70 years. At baseline, 12% of the participants had a history of malignancy, which is notably lower than the approximately 20% malignancy prevalence reported for the general Japanese population of the same age group [18]. This discrepancy is likely attributable to the study’s exclusion criteria, which excluded individuals under active treatment for malignancy. Furthermore, a study of 7415 Japanese CKD patients with a similar mean age reported a new malignancy incidence of 2.4–3.5% over six months [19]. Compared to this report, the malignancy incidence observed in our study appears to be lower. Given these considerations, the malignancy incidence in our cohort may be underestimated, and the actual risk in the general CKD population may be higher.

Several studies recommend against the proactive use of ESAs in the presence of malignancy [20–22]. While ESA use is generally avoided in diagnosed cases, our findings suggest that ESA hyporesponsiveness may be indicative of an undiagnosed malignancy, underscoring the need to evaluate the underlying causes of poor ESA response. With the advent of hypoxia-inducible factor prolyl hydroxylase (HIF-PH) inhibitors, treatment options for ESA-hyporesponsive anemia have expanded, and randomized trials have demonstrated efficacy in raising hemoglobin [23–25]. However, contemporary guidance emphasizes that ESA hyporesponsiveness should prompt a systematic search for reversible causes, such as inflammation, iron disorders, malignancy, and other relevant conditions [13]. Accordingly, before switching to a HIF-PH inhibitor, it is important that clinicians evaluate such underlying conditions so that anemia therapy is tailored appropriately and important comorbidities are not overlooked.

This study has several limitations. First, the timing and stage at which the malignancies were detected were unknown, which may have influenced the relationship between ESA hyporesponsiveness and the development of malignancy. Second, the cohort predominantly consisted of Japanese individuals, potentially limiting the generalizability of our findings to other racial or ethnic populations. Therefore, the external validity of these results in more diverse populations remains uncertain. Future studies in ethnically and racially diverse cohorts could help validate the broader applicability of our findings.

Despite these limitations, our results demonstrated a significant association between initial ESA hyporesponsiveness, as well as subsequent decline in responsiveness and the subsequent development of malignancy in patients with NDD-CKD. Monitoring ESA responsiveness could provide valuable insights for identifying patients at potential risk for malignancy.

## Supplementary Information

Below is the link to the electronic supplementary material.

Supplementary file1 (DOCX 584 KB)

## Data Availability

The R code used in this study is available on request to the corresponding author. Access to the data required the approval of Niigata University and Kyowa Kirin.
