# Supplementary material for: Erythropoiesis-stimulating agent hyporesponsiveness and malignancy development in patients with non-dialysis chronic kidney disease: a prospective cohort study
Source: Clin Exp Nephrol. 2025 Oct 27;30(2):240–7. doi: 10.1007/s10157-025-02769-7 (PMC12886346; doi:10.1007/s10157-025-02769-7)
Supplement: Supplementary file 1 — Supplementary file1 (DOCX 584 KB) [file 10157_2025_2769_MOESM1_ESM.docx]

**Supplemental Materials**

**Supplemental Tables**

Supplemental Table S1. The list of new malignancies by class.

Supplemental Table S2. ESA responsiveness trends and the occurrence of new malignancies in the two latent classes using 36 weeks as an alternative censoring point to the primary 96-week.

Supplemental Table S3. ESA responsiveness trends and the occurrence of new malignancies in the two latent classes using 60 weeks as an alternative censoring point to the primary 96-week.

**Supplemental Figure**

Supplemental Fig. S1. Trajectories of ERI-1B in the two latent classes using 36 weeks as an alternative censoring point to the primary 96-week.

Supplemental Fig. S2. Trajectories of ERI-1B in the two latent classes using 60 weeks as an alternative censoring point to the primary 96-week.

Supplemental Fig. S3. ESA responsiveness stratified by class of ERI-1B trajectory (up to 36 weeks) and incident malignancy

Supplemental Fig. S4. ESA responsiveness stratified by class of ERI-1B trajectory (up to 60 weeks) and incident malignancy

**Supplemental Table S1. The list of new malignancies by class.**

| **Types of malignancy** | Overall  N=44 | Class 1  N=28 | Class 2  N=16 | Fisher's exact test |
| --- | --- | --- | --- | --- |
| Colorectal cancer | 9 | 2 | 7 | p<0.001 |
| Stomach cancer | 6 | 6 | 0 | p=0.3 |
| Prostate cancer | 4 | 3 | 1 | p=1.0 |
| Lung cancer | 3 | 2 | 1 | p=0.5 |
| Bladder cancer | 3 | 2 | 1 | p=0.5 |
| Liver cancer | 2 | 1 | 1 | p=0.4 |
| Laryngeal cancer | 2 | 1 | 1 | p=0.4 |
| Malignant lymphoma | 3 | 2 | 1 | p=0.5 |
| Skin cancer | 3 | 2 | 1 | p=0.5 |
| Esophageal cancer | 1 | 1 | 0 | p=1.0 |
| Renal and urinary tract cancer | 1 | 1 | 0 | p=1.0 |
| Gallbladder cancer | 1 | 0 | 1 | p=0.2 |
| Thyroid cancer | 1 | 1 | 0 | p=1.0 |
| Parathyroid cancer | 1 | 1 | 0 | p=1.0 |
| Myelodysplastic syndrome | 1 | 0 | 1 | p=0.2 |
| Tongue cancer | 1 | 1 | 0 | p=1.0 |
| Submucosal tumours | 1 | 1 | 0 | p=1.0 |
| Adenocarcinoma | 1 | 1 | 0 | p=1.0 |

**Supplemental Table S2. ESA responsiveness trends and the occurrence of new malignancies in the two latent classes using 36 weeks as an alternative censoring point to the primary 96-week.**

|  | Number of patients | Number of new malignancies | Incident rate  (/100 Pearson years)  (95% CI) | Hazard ratio  (95% CI) | Adjusted Hazard ratio  (95% CI) † |
| --- | --- | --- | --- | --- | --- |
| Class 1 (good response group) | 1,340 | 32 | 1.29 (0.88-1.82) | Ref | Ref |
| Class 2 (poor response group) | 301 | 12 | 2.49 (1.29-4.36) | 1.90 (0.98-3.70) | 1.71 (0.88-3.36) |

Class classification was based on the joint latent class mixed model using the ERI-1B transition pattern from 12 to 36 weeks of ESA administration.

ERI-1B was calculated as [DA dose (μg) / Hemoglobin concentration (g/dL)].

† Analyses were adjusted for age, history of malignancy, smoking habits, diabetes status, baseline ferritin levels, high-sensitive C-reactive protein, serum albumin, and estimated glomerular filtration rate.

Abbreviations: ESA, Erythropoiesis-stimulating agent; ERI, erythropoietin resistance index; CI, confidence interval.

**Supplemental Table S3. ESA responsiveness trends and the occurrence of new malignancies in the two latent classes using 60 weeks as an alternative censoring point to the primary 96-week.**

|  | Number of patients | Number of new malignancies | Incident rate  (/100 Pearson years)  (95% CI) | Hazard ratio  (95% CI) | Adjusted Hazard ratio  (95% CI) † |
| --- | --- | --- | --- | --- | --- |
| Class 1 (good response group) | 1,222 | 27 | 1.19 (0.78-1.73) | Ref | Ref |
| Class 2 (poor response group) | 419 | 17 | 2.43 (1.42-3.89) | 2.01 (1.09-3.68) | 1.91 (1.03-3.52) |

Class classification was based on the joint latent class mixed model using the ERI-1B transition pattern from 12 to 60 weeks of ESA administration.

ERI-1B was calculated as [DA dose (μg) / Hemoglobin concentration (g/dL)].

† Analyses were adjusted for age, history of malignancy, smoking habits, diabetes status, baseline ferritin levels, high-sensitive C-reactive protein, serum albumin, and estimated glomerular filtration rate.

Abbreviations: ESA, Erythropoiesis-stimulating agent; ERI, erythropoietin resistance index; CI, confidence interval.

**Supplemental Fig. S1. Trajectories of ERI-1B in the two latent classes using 36 weeks as an alternative censoring point to the primary 96-week.**

**
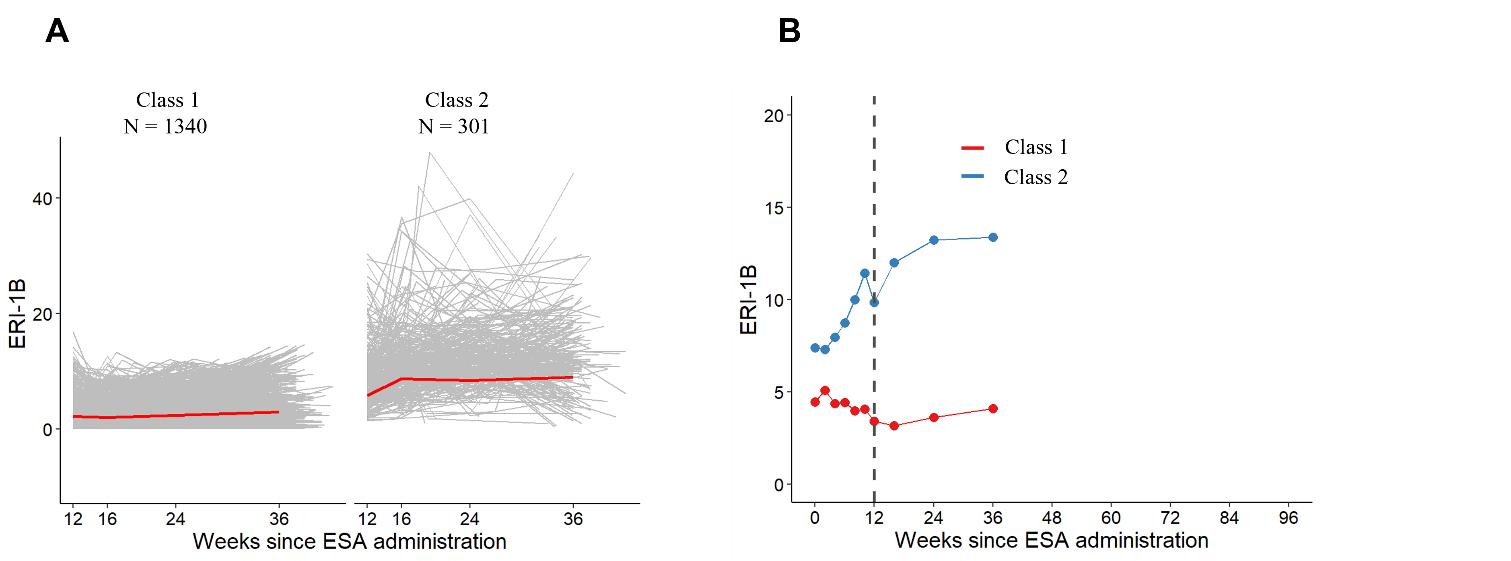
**

b

a

(a) Trajectories of ERI-1B from 12 to 36 weeks after the initiation of ESA administration. The gray lines indicate the ERI-1B trends for individual cases. The red line indicates average ERI-1B values. (b) Changes in the adjusted mean ERI-1B values using the MMRM method in the two latent classes from initiation of ESA administration to 36 weeks. Changes from the initiation of ESA administration to 12 weeks were not reflected in the class classification.

Abbreviations: ERI, erythropoietin resistance index; ESA, Erythropoiesis-stimulating agents.

**Supplemental Fig. S2. Trajectories of ERI-1B in the two latent classes using 60 weeks as an alternative censoring point to the primary 96-week.**

b

a

**
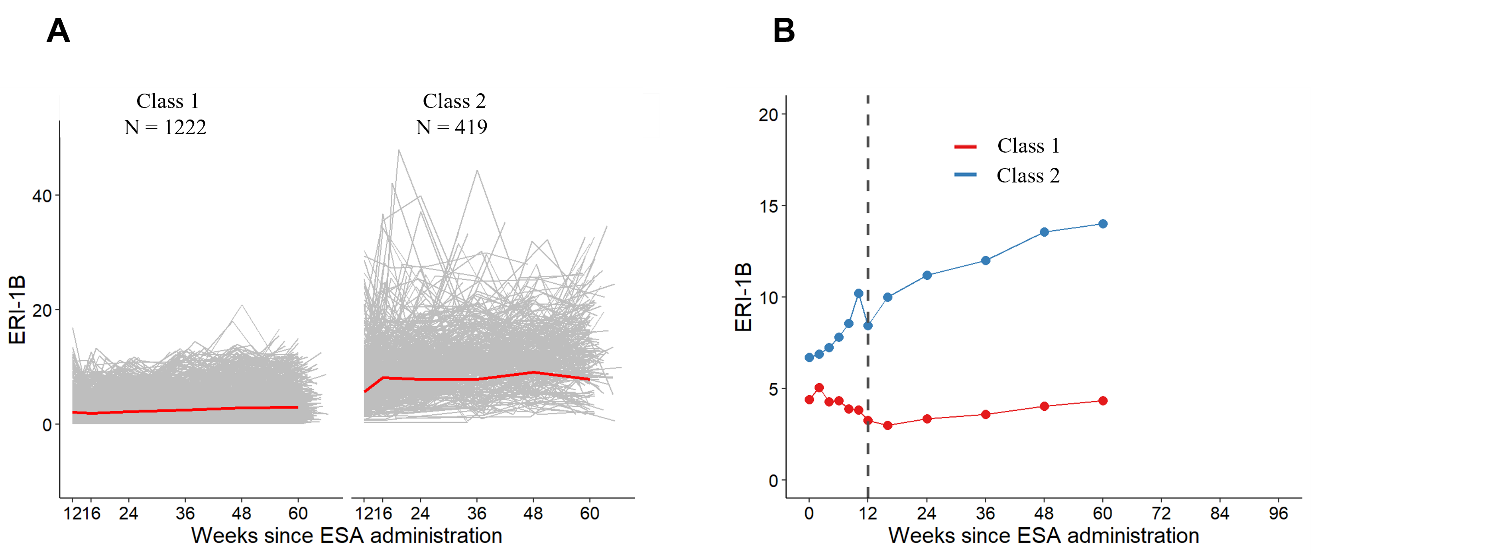
**

(a) Trajectories of ERI-1B from 12 weeks after the initiation of ESA administration to 60 weeks. The gray lines indicate the ERI-1B trends for individual cases. The red line indicates average ERI-1B values. (b) Changes in the adjusted mean ERI-1B values from the initiation of ESA administration to 60 weeks, using the MMRM method in the two latent classes. Changes from the initiation of ESA administration to 12 weeks were not reflected in the class classification.

Abbreviations: ERI, erythropoietin resistance index; ESA, Erythropoiesis-stimulating agents.

**Supplemental Fig. S3. ESA responsiveness stratified by class of ERI-1B trajectory (up to 36 weeks) and incident malignancy**

**
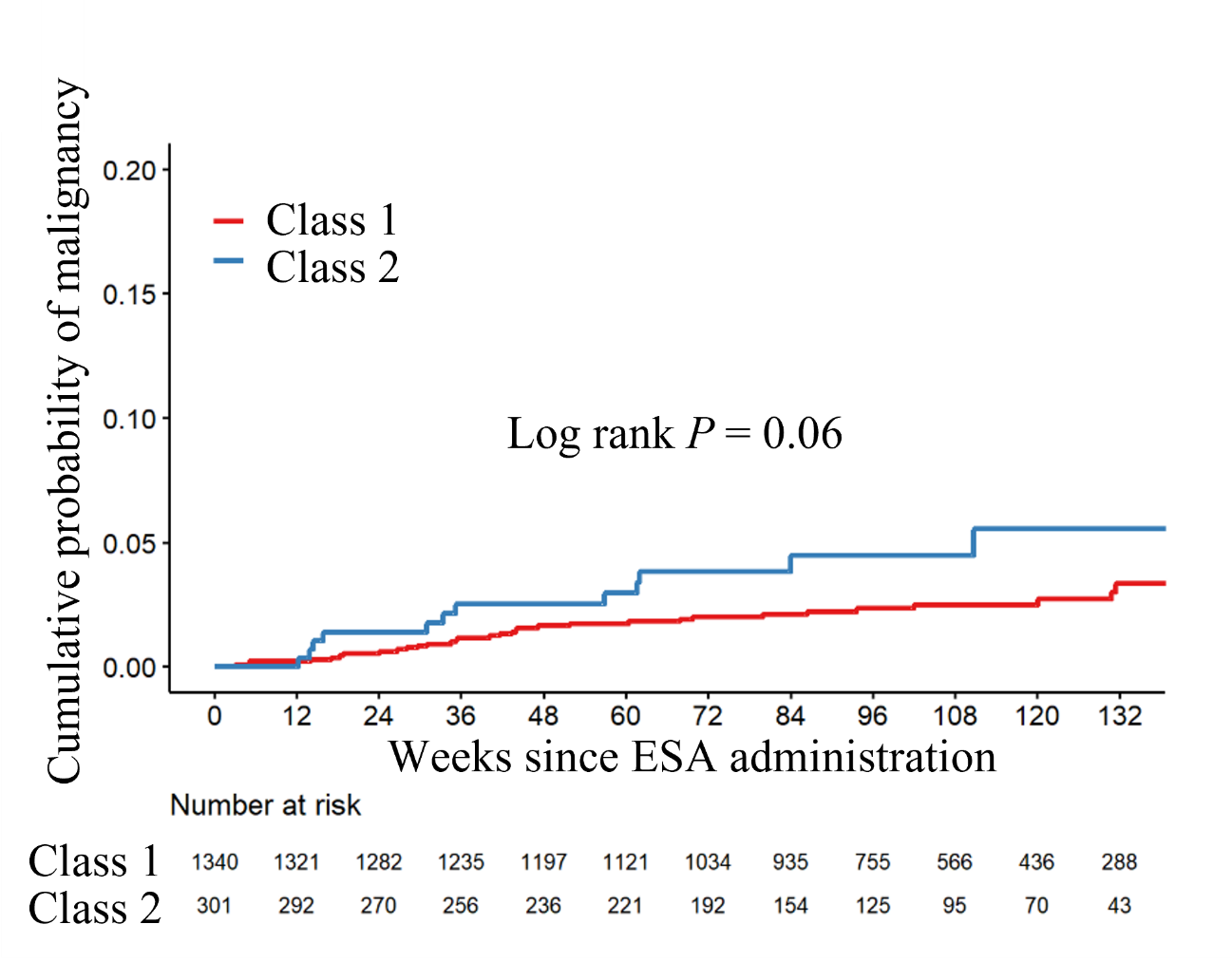
**

Class 2 patients (poor response group) had a higher incidence of malignancy than Class 1 patients (good response group), although the difference was not statistically significant (log-rank *P*=0.06).

Abbreviations: ESA, Erythropoiesis-stimulating agents; ERI, erythropoietin resistance index.

**Supplemental Fig. S4. ESA responsiveness stratified by class of ERI-1B trajectory (up to 60 weeks) and incident malignancy**

**
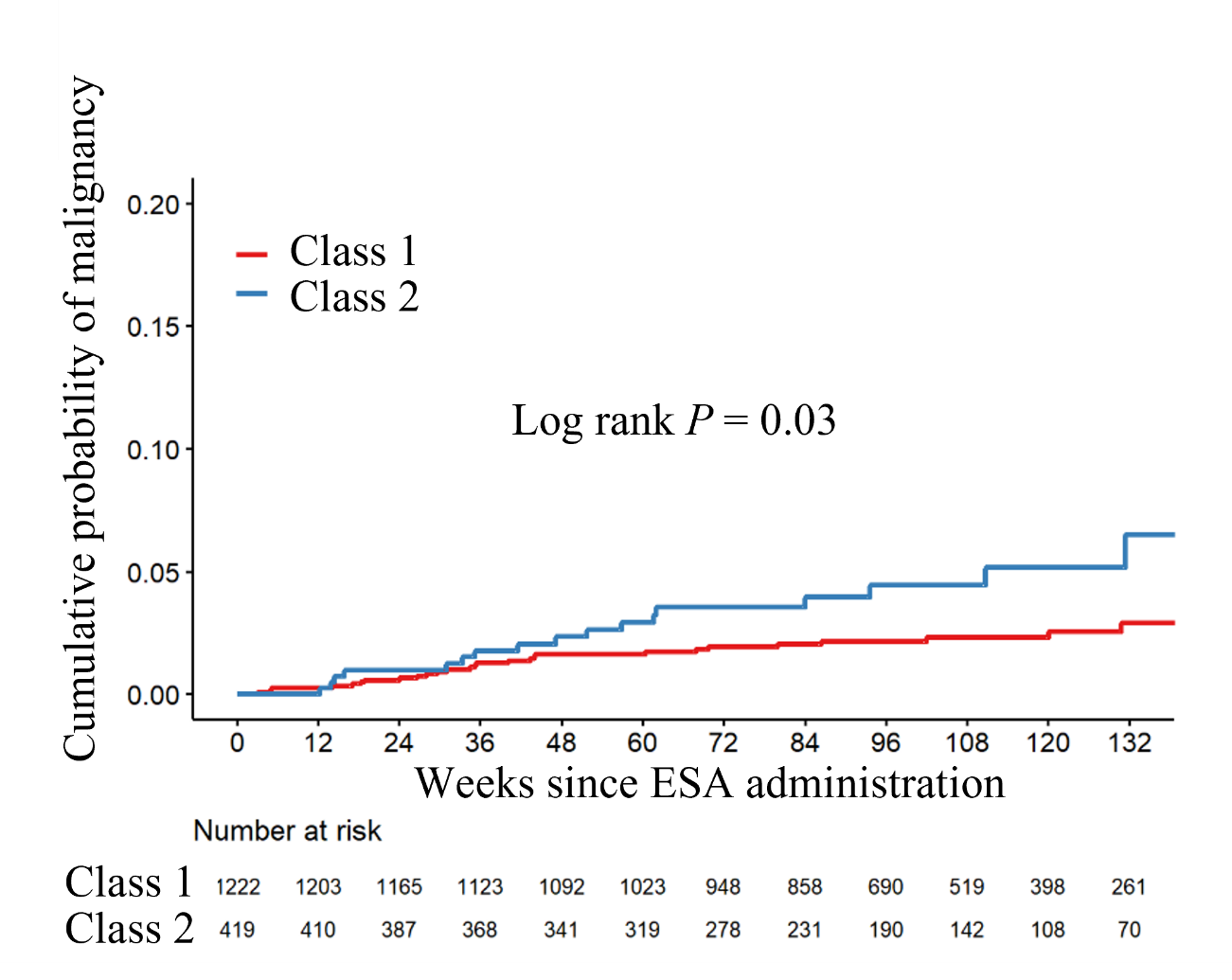
**

Class 2 (poor response group) had a significantly higher incidence of malignancy than class 1 (good response group) (log-rank, *P*=0.03).

Abbreviations: ESA, Erythropoiesis-stimulating agents; ERI, erythropoietin resistance index.
